# 无机焦磷酸酶对急性B淋巴细胞白血病异常幼稚细胞增殖、凋亡的影响

**DOI:** 10.3760/cma.j.cn121090-20251103-00499

**Published:** 2026-07

**Authors:** 晨曦 胡, 旭华 张, 小姣 汪, 佳佳 代, 帆 张, 旭东 魏, 晓东 吕

**Affiliations:** 郑州大学附属肿瘤医院（河南省肿瘤医院），郑州 450003 Affiliated Cancer Hospital of Zhengzhou University/Henan Cancer Hospital, Zhengzhou 450003, China

**Keywords:** 无机焦磷酸酶, 急性B淋巴细胞白血病, 细胞增殖, 细胞凋亡, Inorganic pyrophosphatase, B-cell acute lymphoblastic leukemia, Cell proliferation, Apoptosis

## Abstract

**目的:**

探讨无机焦磷酸酶（inorganic pyrophosphatase，PPA1）对急性B淋巴细胞白血病（B-cell acute lymphoblastic leukemia，B-ALL）异常幼稚淋巴细胞增殖、凋亡的影响，评估PPA1作为肿瘤标志物的潜力。

**方法:**

生物信息学分析PPA1基因特性。收集患者骨髓标本，经CD19和CD10免疫磁珠分选异常幼稚B淋巴细胞，以正常细胞为对照，采用qPCR及Western blot法检测PPA1表达。体外培养B-ALL细胞系NALM-6和HAL-01细胞，分别加入重组PPA1蛋白（recombinant PPA1，rPPA1）或PPA1单抗，采用CCK-8实验和流式细胞术检测细胞增殖、凋亡、线粒体膜电位及细胞周期变化。构建GV492-PPA1过表达质粒转染细胞，检测PPA1过表达的影响。设计shRNA构建GV644-shPPA1重组质粒，慢病毒转染抑制细胞PPA1表达，观察对肿瘤细胞的影响，并检测PI3K/AKT通路关键蛋白及凋亡相关因子表达水平。

**结果:**

B-ALL患者异常幼稚淋巴细胞中PPA1转录与表达水平分别为正常细胞的3.75～14.89倍和1.25～3.79倍。rPPA1可促进NALM-6与HAL-01细胞增殖，10 µg/ml处理72 h后，增殖率分别提高29.97％和20.96％；20 µg/ml时提高48.40％和58.08％。PPA1单抗（1∶2 000、1:1 000、1:500）显著抑制增殖，NALM-6细胞增殖抑制率分别为19.53％、46.90％和49.42％，HAL-01细胞分别为22.29％、52.41％和58.35％。PPA1单抗（1:2 000、1:1 000）促进细胞凋亡，使NALM-6细胞凋亡率由10.01％分别升至20.17％和29.11％，HAL-01细胞由12.65％升至18.53％和31.80％。经相同稀释度PPA1单抗处理后，JC-1单体比例显著增加，NALM-6细胞为对照组的4.08和6.49倍，HAL-01细胞为3.99和6.42倍，表明线粒体膜电位降低，同时将细胞周期阻滞于G_0_/G_1_期。过表达PPA1并培养48和72 h后，NALM-6细胞增殖率提高25.05％和26.14％，HAL-01细胞提高26.14％和29.25％；抑制PPA1表达则抑制增殖，NALM-6细胞抑制率为19.77％（48 h）和28.99％（72 h），HAL-01细胞为20.90％（72 h）。qPCR和Western blot检测显示，抑制PPA1可降低PI3K/AKT通路磷酸化水平，调控MDM2、P53及凋亡相关因子表达。

**结论:**

PPA1与B-ALL异常幼稚淋巴细胞增殖发育关系密切，抑制PPA1表达可抑制肿瘤细胞增殖，促进凋亡，具有作为肿瘤防治新靶标的潜力。

## Abstract (en)

**Objective:**

To investigate the role of inorganic pyrophosphatase（PPA1）in the proliferation of abnormal naive lymphocytes in B-cell acute lymphoblastic leukemia（B-ALL）and evaluate its potential as a tumor marker.

**Methods:**

Bioinformatics analysis predicted the characteristics of the PPA1 gene. Bone marrow specimens were collected from patients with B-ALL, and abnormal immature B lymphocytes were isolated by CD19 and CD10 immunomagnetic beads, with normal cells as controls. PPA1 expression was detected with quantitative polymerase chain reaction（qPCR）and Western blot. B-ALL cell lines NALM-6 and HAL-01 were cultured *in vitro*, and recombinant PPA1 protein（rPPA1）or PPA1 monoclonal antibody was added to assess changes in cell proliferation, apoptosis, mitochondrial membrane potential, and cell cycle. The GV492-PPA1 overexpression plasmid was constructed and transfected into cells to assess the effect of PPA1 overexpression. shRNA was designed to construct the GV644-shPPA1 recombinant plasmid, and lentiviral transduction was performed to inhibit PPA1 expression. The impact on tumor cells was observed, and the expression levels of key proteins in the PI3K/AKT pathway and apoptosis-related factors were detected.

**Results:**

In abnormal immature B lymphocytes from patients with B-ALL, the transcription and expression levels of PPA1 were 3.75–14.89 times and 1.25–3.79 times those of normal cells, respectively. rPPA1 promoted NALM-6 and HAL-01 cell proliferation, with increased proliferation rates of 29.97％ and 20.96％ after treatment with 10 µg/ml for 72 h and 48.40％ and 58.08％ at 20 µg/ml, respectively. PPA1 monoclonal antibody（1:2 000, 1:1 000, 1:500）significantly inhibited proliferation at 19.53％, 46.90％, and 49.42％ for NALM-6 cells and 22.29％, 52.41％, and 58.35％ for HAL-01 cells, respectively. PPA1 monoclonal antibody（1:2 000, 1:1 000）induced apoptosis, increasing the apoptosis rate of NALM-6 cells from 10.01％ to 20.17％ and 29.11％, and that of HAL-01 cells from 12.65％ to 18.53％ and 31.80％. After treatment with the same PPA1 monoclonal antibody dilutions, the proportion of JC-1 monomers significantly increased, reaching 4.08- and 6.49-fold of the control in NALM-6 cells, and 3.99- and 6.42-fold in HAL-01 cells, indicating a decrease in mitochondrial membrane potential, whereas the cell cycle was arrested at the G_0_/G_1_ phase. Overexpression of PPA1 for 48 and 72 h increased the proliferation rate of NALM-6 cells by 25.05％ and 26.14％, and that of HAL-01 cells by 26.14％ and 29.25％, respectively. PPA1 expression inhibition suppressed proliferation, with inhibition rates of 19.77％（48 h）and 28.99％（72 h）for NALM-6 cells, and 20.90％（72 h）for HAL-01 cells. qPCR and Western blot results revealed that PPA1 inhibition reduced the phosphorylation level of the PI3K/AKT pathway and regulated the expression of MDM2, P53, and apoptosis-related factors.

**Conclusion:**

PPA1 is closely associated with the proliferation and development of abnormal immature lymphocytes in B-ALL. PPA1 expression inhibition suppresses abnormal cell proliferation and promotes apoptosis, indicating that PPA1 has potential as a new target for tumor prevention and treatment.

急性B淋巴细胞白血病（B-cell acute lymphoblastic leukemia，B-ALL）是起源于造血干细胞的恶性克隆性疾病，以骨髓中原始及幼稚B淋巴细胞异常增生、聚集并抑制正常造血为特征。成人B-ALL患者治疗效果相对欠佳，虽70％～90％患者可获得完全缓解，然而获得缓解的患者中仍有30％～50％呈可检测残留病（MRD）阳性，MRD阳性是导致该病复发的重要原因[1]–[2]。该病在治疗过程中易产生耐药性，复发率高达40％，严重影响患者预后及生活质量。抑制原始及幼稚B淋巴细胞异常增殖是治疗B-ALL的关键，因此探寻能够有效抑制异常幼稚B淋巴细胞生长的基因靶点，可为临床提供新的治疗策略[3]–[4]。

无机焦磷酸酶（inorganic pyrophosphatase，PPA1），简称焦磷酸酶，在原核和真核生物中普遍存在，参与核酸聚合、辅酶合成、氨基酸及脂肪酸活化等众多生化反应。PPA1可催化底物焦磷酸盐（PPi）水解成两分子的磷酸盐（Pi），从而调控细胞内PPi与Pi的浓度平衡[5]–[6]。细胞快速分裂需要持续的磷酸盐供应，磷酸盐是核酸、磷脂和ATP等高能代谢物合成的必需原料[7]。研究表明，PPi在生物能量代谢中具有重要作用，特定条件下可替代ATP为细胞供能。PPA1促进细胞代谢过程产生的PPi水解，可为细胞磷酸化反应持续提供正磷酸。若PPA1缺失，PPi水解速率可降低至原来的1/10^10^［8]。此外，PPA1催化水解过程中产生的能量可促进辅酶合成及其他生化反应的进行，如脂肪和氨基酸活化[9]。目前，PPA1在B-ALL异常幼稚细胞增殖与发育中的作用尚未见报道。因此，本研究旨在探索PPA1在B-ALL患者白血病细胞中的表达水平，分析其过表达及沉默对B-ALL细胞株增殖、凋亡的影响，为B-ALL临床治疗提供新思路。

## 材料与方法

1. 病例资料：以2022年7月至2023年7月于河南省肿瘤医院血液科就诊的12例B-ALL患者为主要研究对象，其中男6例，女6例，疾病诊断和分型参考《中国成人急性淋巴细胞白血病诊断与治疗指南（2024年版）》[2]。另随机选取6例因不明原因发热、免疫性血小板减少、淋巴结肿大、血象异常等原因就诊，最终确诊为非造血系统疾病，且经骨髓形态学及流式细胞术检测均未见异常的患者作为正常对照。所有入组患者年龄范围为20～60岁。本研究经医院伦理委员会审批通过（批件号：2019337），所有患者均签署知情同意书。

2. 生物信息学分析及进化树的建立：ProtParam分析等电点（pI）并预测PPA1蛋白相对分子质量；NCBI在线预测PPA1蛋白结构域及其活性位点；利用Interpro预测PPA1信号肽、亲疏水性及跨膜域；DNAstar和SWISS-MODEL分析建立PPA1蛋白二三级结构模型；Mega 7.0软件用于不同物种PPA1氨基酸序列进化树的构建[10]–[11]。

3. 异常/正常幼稚B淋巴细胞筛选：采集B-ALL患者及正常对照者骨髓标本2～5 ml，乙二胺四乙酸（EDTA）抗凝，淋巴细胞Ficoll分离液提取单个核细胞后采用德国美天旎生物技术有限公司CD19和CD10免疫磁珠过柱分选出异常/正常幼稚B淋巴细胞[12]。

4. 核酸提取及细胞可溶性蛋白的制备：Trizol法提取细胞RNA，反转录为cDNA，经NanoDrop2000检测浓度后于−20 °C保存。使用细胞蛋白快速提取试剂盒制备细胞可溶性蛋白，于−20 °C保存。

5. 患者异常幼稚B淋巴细胞PPA1转录及蛋白表达水平检测：以GAPDH为内参基因，根据PPA1基因序列设计qPCR引物，上游引物：5′-CTCTCTCCTTGTCAGTCGGC-3′，下游引物：5′-GAAGACTCGGTACTCCAGGG-3′，正常幼稚B淋巴细胞为对照，采用qPCR检测患者异常幼稚B淋巴细胞中PPA1基因转录水平[13]，每个样本设置3个复孔，以GAPDH为内参基因。采用2^−∆∆Ct^法计算相对表达量，以正常对照组表达水平为参照进行归一化。同时，以PPA1单抗为一抗进行Western blot检测细胞中PPA1蛋白表达情况[14]。

6. 细胞培养：本研究选用NALM-6与HAL-01两种B-ALL细胞系进行体外实验。于超净台中将冻存细胞置于37 °C水浴快速融化，无菌吸管吸出细胞悬液，加入5 ml含10％胎牛血清（FBS）的RPMI 1640完全培养基，混匀后置于37 °C、5％ CO_2_细胞培养箱中复苏。培养24 h后换液[15]。待细胞生长至汇合度约90％时进行传代培养。

7. 重组PPA1蛋白（rPPA1）的获得及蛋白活性检测：将PPA1全长编码序列（由上海生工生物工程股份有限公司合成）经BamH Ⅰ和Pst Ⅰ双酶切后，克隆至经相同酶切线性化的pQE-80L表达载体。连接产物转化至大肠杆菌DH5α感受态细胞，挑取阳性克隆进行测序验证。将序列正确的重组质粒PPA1/pQE-80L转化至表达宿主大肠杆菌BL21。挑取单菌落接种于含氨苄青霉素（100 µg/ml）的LB液体培养基中，加入1.0 mmol/L IPTG，于37 °C诱导6 h。离心收集菌体，超声裂解后，采用Ni-NTA柱亲和纯化rPPA1蛋白，SDS-PAGE鉴定纯化产物[6]。纯化后的rPPA1蛋白活性采用钼蓝比色法测定，反应体系（总体积200 µl）包含：100 mmol/L Tris-HCl缓冲液（pH 7.5）、5 mmol/L MgCl_2_、1 mmol/L Na_4_P_2_O_7_（PPi），以及适量rPPA1蛋白（0.5 µg）。在55 °C水浴中孵育20 min后加入1 ml 200 mmol/L甘氨酸-盐酸缓冲液（pH 3.0）终止反应。随后加入125 µl 1％ Na₂MoO₄（溶于25 mmol/L H₂SO₄）和2.5％ SnCl₂溶液（溶于甘油），混匀后于37 °C静置15 min。酶标仪检测620 nm波长处吸光度（*A*_620_）值[6],[16]。

8. rPPA1蛋白对NALM-6和HAL-01细胞增殖的影响：取对数期NALM-6与HAL-01细胞接种至96孔板（5×10^4^个/孔），每孔加入含10％ FBS的RPMI 1640完全培养基（总体积100 µl）。在培养基中加入rPPA1（0、5、10、20 µg/ml），每组设置3个复孔，同时设置无细胞空白对照孔。分别培养24、36、48、72 h，然后每孔加入10 µl CCK-8溶液，混匀于37 °C避光孵育2～4 h，酶标仪检测*A*_450_值。细胞增殖率（％）＝（实验组*A*_450_−空白组*A*_450_）/（对照组*A*_450_−空白组*A*_450_）×100％。

9. PPA1单抗及NaF抑制剂对NALM-6和HAL-01细胞增殖的影响：取对数期NALM-6与HAL-01细胞接种至96孔板（5×10^4^个/孔），每孔加入含10％ FBS的RPMI 1640完全培养基（总体积100 µl）。于细胞培养基中分别加入PPA1单抗（稀释度为1∶3 000、1∶2 000、1∶1 000、1∶500）和抑制剂NaF（1、3、5、7 µmol/L），每组设置3个复孔，同时设置无细胞空白对照孔和实验对照孔（即未经PPA1单抗和NaF抑制剂处理的细胞，加入等体积PBS）。培养24 h后每孔加入10 µl CCK-8溶液，混匀后于37 °C避光孵育2～4 h，酶标仪检测*A*_450_值，计算细胞增殖率。

10. 细胞凋亡实验：取对数期NALM-6与HAL-01细胞接种于24孔板（2×10^5^个/孔），每孔加入含10％ FBS的RPMI 1640完全培养基（总体积500 µl）。分别设置未处理细胞对照组、PPA1单抗组（分别以1∶1 000、1∶2 000和1∶3 000稀释）和NaF抑制剂组（1、3、5、7 µmol/L）。培养24 h后收集细胞，PBS洗涤3次，每管加入500 µl 1×结合缓冲液重悬细胞，依次加入5 µl Annexin Ⅴ-AbFluor FITC和5 µl PI，轻轻混匀，于室温避光孵育15 min。孵育结束后，流式细胞仪检测，每管采集至少1×10⁴个细胞，使用FITC通道（FL1）检测Annexin Ⅴ阳性细胞，PE通道（FL2）检测PI阳性细胞。实验独立重复3次。细胞凋亡率（％）＝早期凋亡率（Annexin Ⅴ⁺/PI⁻）+晚期凋亡率（Annexin Ⅴ⁺/PI⁺）。

11. 线粒体膜电位及细胞周期检测：线粒体膜电位下降是细胞凋亡的早期标志，线粒体膜电位较高时JC-1易形成聚合物，聚集在线粒体基质中，而线粒体膜电位降低时，JC-1不能聚集在基质中，以单体形式存在。采用JC-1线粒体膜电位检测试剂盒（北京索莱宝科技有限公司）检测线粒体膜电位。取对数生长期的NALM-6与HAL-01细胞，接种于24孔板中（2×10^5^个/孔），每孔加入含10％ FBS的RPMI 1640完全培养基（总体积500 µl）。分别设置PPA1单抗组（稀释度1∶1 000、1∶2 000和1∶3 000）和未处理对照组（加入等体积PBS），每组设3个复孔，培养24 h后收集细胞，500 µl完全培养基重悬。然后加入500 µl JC-1染色工作液，混匀后于37 °C、5％ CO_2_培养箱中避光孵育20 min。孵育后收集细胞，1× JC-1染色缓冲液洗涤2次后加入500 µl 1× JC-1染色缓冲液重悬细胞，经流式细胞仪检测，本实验独立重复3次。采用PI单染法检测细胞周期，将对数期NALM-6与HAL-01细胞接种于24孔板（2×10^5^个/孔），加入含10％ FBS的RPMI 1640培养基。设置PPA1单抗组（1∶1 000和1∶2 000）和未处理对照组，每组设3个复孔。培养24 h后收集细胞，预冷PBS洗涤，逐滴加入预冷70％乙醇，4 °C固定过夜。PBS洗涤后，加入PI染色液（50 µg/ml PI、100 µg/ml RNase A、0.1％ Triton X-100），37 °C避光孵育30 min后经流式细胞仪检测，Kaluza软件分析周期分布。

12. PPA1过表达对NALM-6和HAL-01细胞增殖的影响：合成PPA1过表达序列并连接至慢病毒GV492载体，构建成GV492-PPA1过表达重组质粒，测序鉴定。将GV492空载体和GV492-PPA1重组质粒及辅助质粒（psPAX2和pMD2.G）共转染293T细胞，转染后48 h和72 h收集病毒上清，浓缩后采用qPCR法测定滴度，两种病毒滴度均为5×10^8^ TU/ml。NALM-6和HAL-01细胞按感染复数（MOI）＝10加入慢病毒（同时加入聚凝胺5 µg/ml），感染12 h后换液。感染48 h后荧光显微镜下观察EGFP表达以确认感染效率；感染72 h后收集细胞，qPCR检测PPA1 mRNA水平，Western blot检测PPA1蛋白水平；CCK-8实验于感染后0、24、36、48、72 h分别检测细胞增殖情况。

13. 抑制PPA1表达对NALM-6和HAL-01增殖的影响：根据PPA1基因（NM_021129.4）设计特异性shRNA，其对应的寡核苷酸序列为：正向：5′-CCGGTGGAAAGTCATTGCCATTAATCTCGAGATTAATGGCAATGACTTTCCATTTTTG-3′；反向：5′-AATTCAAAAATGGAAAGTCATTGCCATTAATCTCGAGATTAATGGCAATGACTTTCCA-3′。将该双链寡核苷酸退火后插入GV644-EGFP穿梭质粒中构建GV644-shPPA1重组质粒。测序鉴定后将GV644空质粒、GV644-shPPA1与辅助质粒（psPAX2、pMD2.G）共转染293T细胞，48 h和72 h收集上清，浓缩后qPCR测定滴度（均为9×10^8^ TU/ml）。NALM-6和HAL-01细胞按MOI＝10加入慢病毒（含5 µg/ml聚凝胺），感染12 h换液。感染48 h荧光观察EGFP确认感染效率，感染72 h后收集细胞进行qPCR和Western blot；CCK-8于感染后0、24、36、48、72 h检测。

14. Western blot检测PI3K/AKT信号通路及凋亡相关因子：收集GV644对照慢病毒和GV644-shPPA1慢病毒感染NALM-6和HAL-01细胞，使用RIPA裂解液（含PMSF及磷酸酶抑制剂）冰上裂解30 min，4 °C离心提取总蛋白，BCA法测定浓度。取20 µg蛋白/泳道经SDS-PAGE分离（5％浓缩胶，12％分离胶），湿转至PVDF膜，于TBST配置的5％脱脂奶粉封闭液中37 °C孵育1 h。封闭结束后TBST洗涤3次，然后进行一抗37 °C孵育2 h，TBST洗涤3遍后加二抗37 °C孵育1 h，二抗结束后TBST洗涤3次。将ECL发光液滴加在PVDF膜上，通过Amersham lmager 600超灵敏多功能成像仪显影，Image J软件进行灰度分析。

15. 统计学处理：所有数据采用SPSS 26.0软件进行统计分析，以均数±标准差表示。所有实验独立重复3次，多组间差异比较采用单因素方差分析。*P*<0.05为差异具有统计学意义。

## 结果

1. PPA1生物学特性在线预测分析结果：经生物信息学分析结果显示：人PPA1基因（Gene ID：NM_021129.4）CDS全长870 bp，编码氨基酸289个；PPA1蛋白相对分子质量预测为32.66×10^3^，pI为5.54，无信号肽、无跨膜区，亲水性较好（**扫描本文首页二维码查阅附图1A～D**）。进化树结果提示人PPA1基因与苏门答腊猩猩进化关系最为密切（**扫描本文首页二维码查阅附图1E**）。蛋白二、三级结构预测结果发现PPA1表面抗原丰富，具有较好的抗原性，含有2个α螺旋和12个β折叠，蛋白表面有金属结合位点、底物结合位点和多肽结合位点，且PPA1常以二聚体或四聚体的形式存在（**扫描本文首页二维码查阅附图2**）。

2. qPCR检测B-ALL患者异常幼稚细胞PPA1转录水平：结果显示，PPA1在12例B-ALL患者异常幼稚淋巴中的转录水平显著升高，为正常对照细胞的3.75～14.89倍，差异具有统计学意义（*F*＝15.34，*P*<0.001）（图1）。

**图1:**
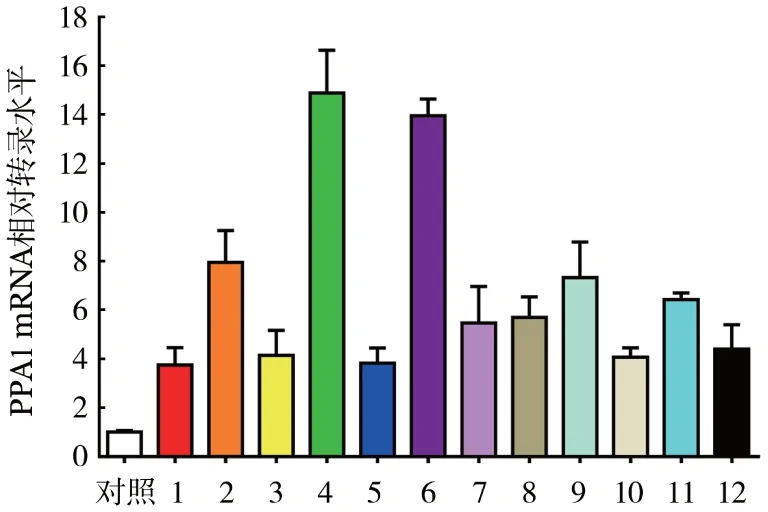
PPA1基因在急性B淋巴细胞白血病患者及正常对照细胞中的相对转录水平

3. Western blot检测B-ALL患者异常幼稚细胞PPA1蛋白水平：Western blot检测结果显示，12例B-ALL患者异常幼稚B淋巴细胞中PPA1蛋白表达水为正常对照组的1.25～3.79倍，差异具有统计学意义（*F*＝173.9，*P*<0.05）（图2），提示PPA1在B-ALL疾病发生发展过程中可能发挥了重要作用。

**图2:**
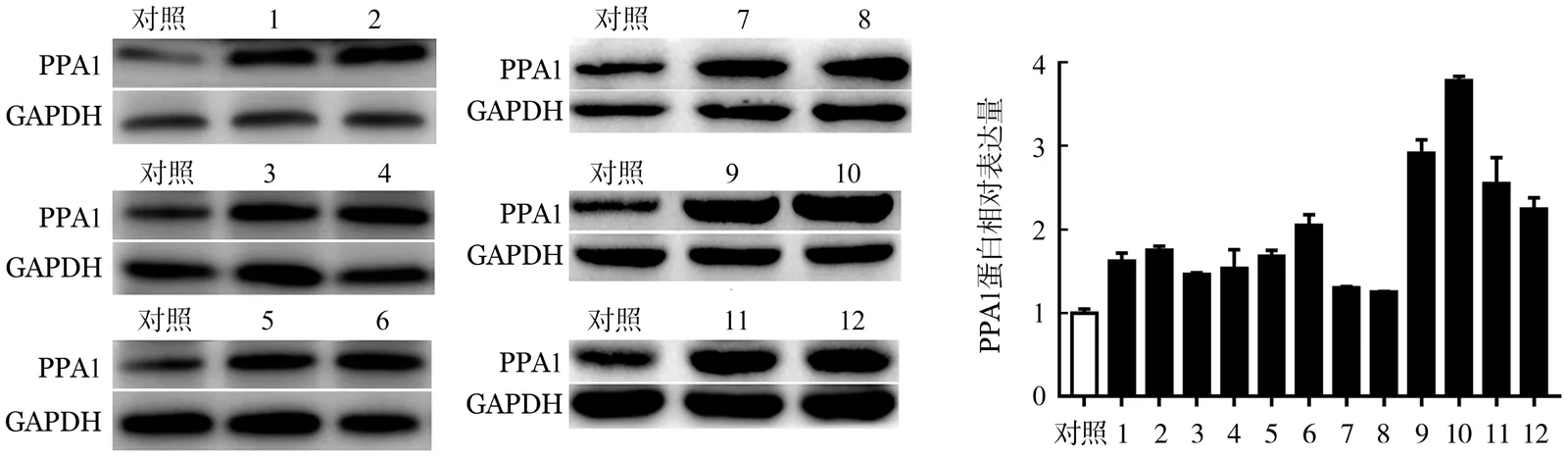
PPA1蛋白在急性B淋巴细胞白血病（B-ALL）患者及正常对照细胞中的相对表达水平（实验重复3次）

4. rPPA1酶活性检测及其对NALM-6与HAL-01细胞增殖的促进作用：为验证PPA1作用的普遍性，本研究联合使用遗传背景稳定的经典B-ALL细胞模型NALM-6及表型存在差异的补充模型HAL-01。SDS-PAGE结果显示，在相对分子质量（25～35）×10^3^可明显观察到rPPA1目的条带，与预测相对分子质量32.66×10^3^一致，说明纯化效果好（图3A）。酶活性检测显示，rPPA1最佳反应条件为50 °C、pH 7.0～7.5及5 mmol/L Mg^2+^（图3B～E）。CCK-8结果表明，不同浓度rPPA1蛋白对细胞增殖的影响存在差异。与对照组相比，5 µg/ml的rPPA1对NALM-6和HAL-01细胞增殖无显著影响。当rPPA1浓度升至10 µg/ml并培养72 h后，NALM-6和HAL-01细胞增殖率显著上升，分别增加了29.97％和20.96％。而将rPPA1浓度进一步增至20 µg/ml并培养72 h后，细胞增殖促进效应更为明显，NALM-6和HAL-01细胞增殖率分别上调了48.40％和58.08％（*P*<0.05）（图3F、G）。

**图3:**
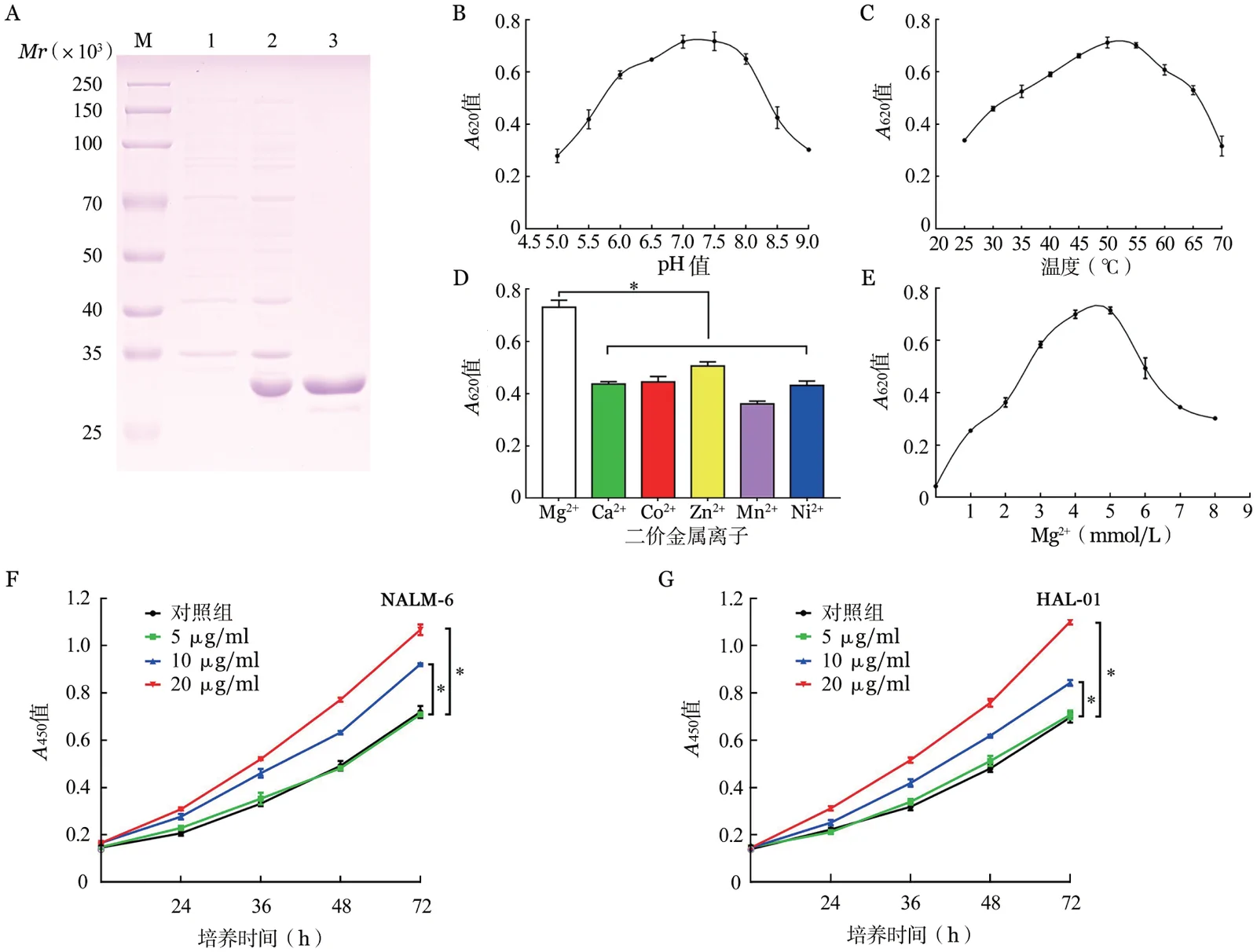
重组PPA1蛋白酶活性检测及对细胞增殖的影响（实验重复3次，*P<0.05） **A** SDS-PAGE检测rPPA1纯化效果（M：Marker；1：未诱导的重组BL21菌；2：诱导后的重组BL21菌；3：纯化后的rPPA1蛋白）；**B～E** 钼蓝比色法检测rPPA1最佳活性表达条件；**F～G** 不同浓度rPPA1对NALM-6和HAL-01细胞增殖的影响

5. PPA1单抗对NALM-6与HAL-01细胞增殖的抑制作用：CCK-8结果显示，与对照组相比，PPA1单抗在1∶2 000、1∶1 000和1∶500稀释度下均可显著抑制NALM-6和HAL-01细胞的增殖（*P*<0.05），且呈浓度依赖性（NALM-6细胞增殖抑制率分别为19.53％、46.90％和49.42％，HAL-01细胞增殖抑制率分别为22.29％、52.41％和58.35％），且1∶2 000稀释条件下PPA1单抗的抑制效果明显弱于1∶1 000和1∶500稀释度（*F*_NALM-6_＝268.67，*F*_HAL-01_＝104.26，*P*<0.05）（图4A）。此外，广谱磷酸酶抑制剂NaF亦可明显抑制这两种细胞的增殖（*P*<0.05）（图4B）。

**图4:**
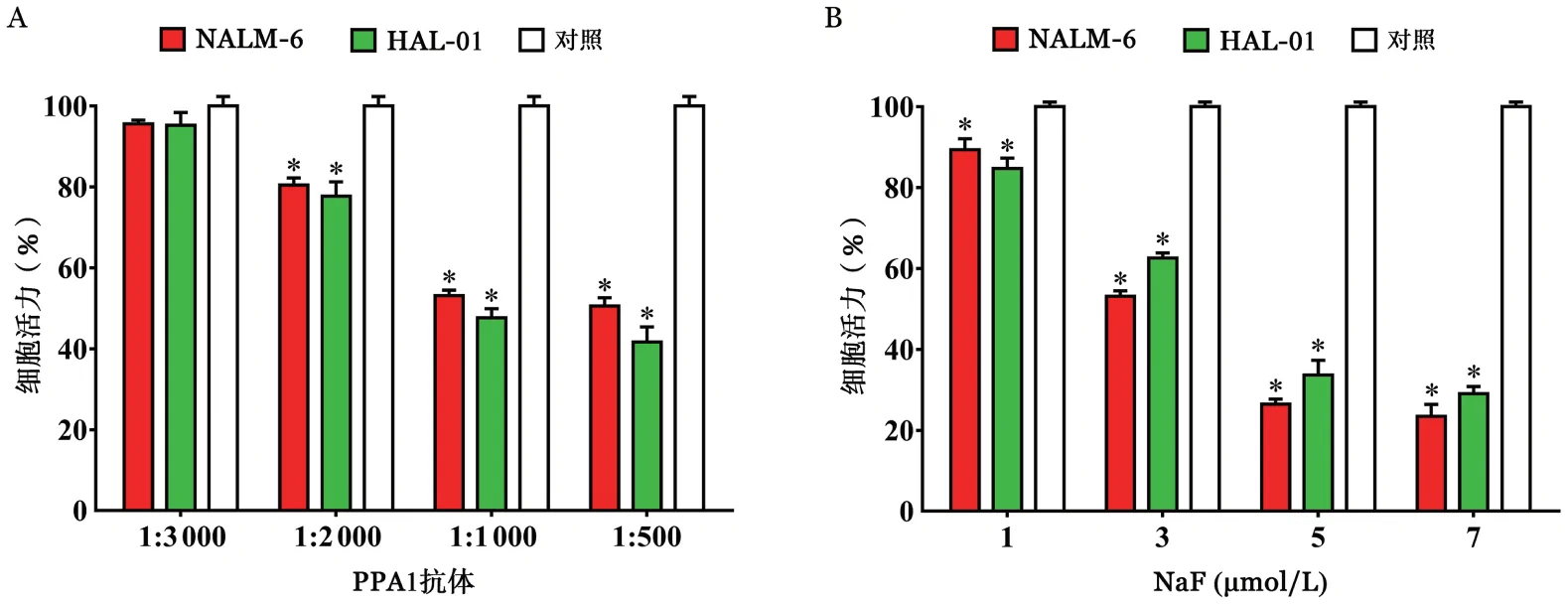
PPA1单抗（Ａ）、NaF（B）对NALM-6与HAL-01细胞增殖的抑制作用（实验重复3次，与对照组比较，*P<0.05）

6. PPA1单抗对NALM-6与HAL-01细胞凋亡的影响：流式细胞术检测结果显示，PPA1 单抗可显著促进NALM-6和HAL-01细胞凋亡，且呈浓度依赖性。与对照组相比，1∶2 000和1∶1 000稀释度的PPA1单抗处理后，NALM-6细胞凋亡率分别升至20.17％和29.11％，HAL-01细胞凋亡率分别升至18.53％和31.80％（*P*<0.05）。NaF亦可显著诱导HAL-01细胞凋亡（*P*<0.05）（图5）。

**图5:**
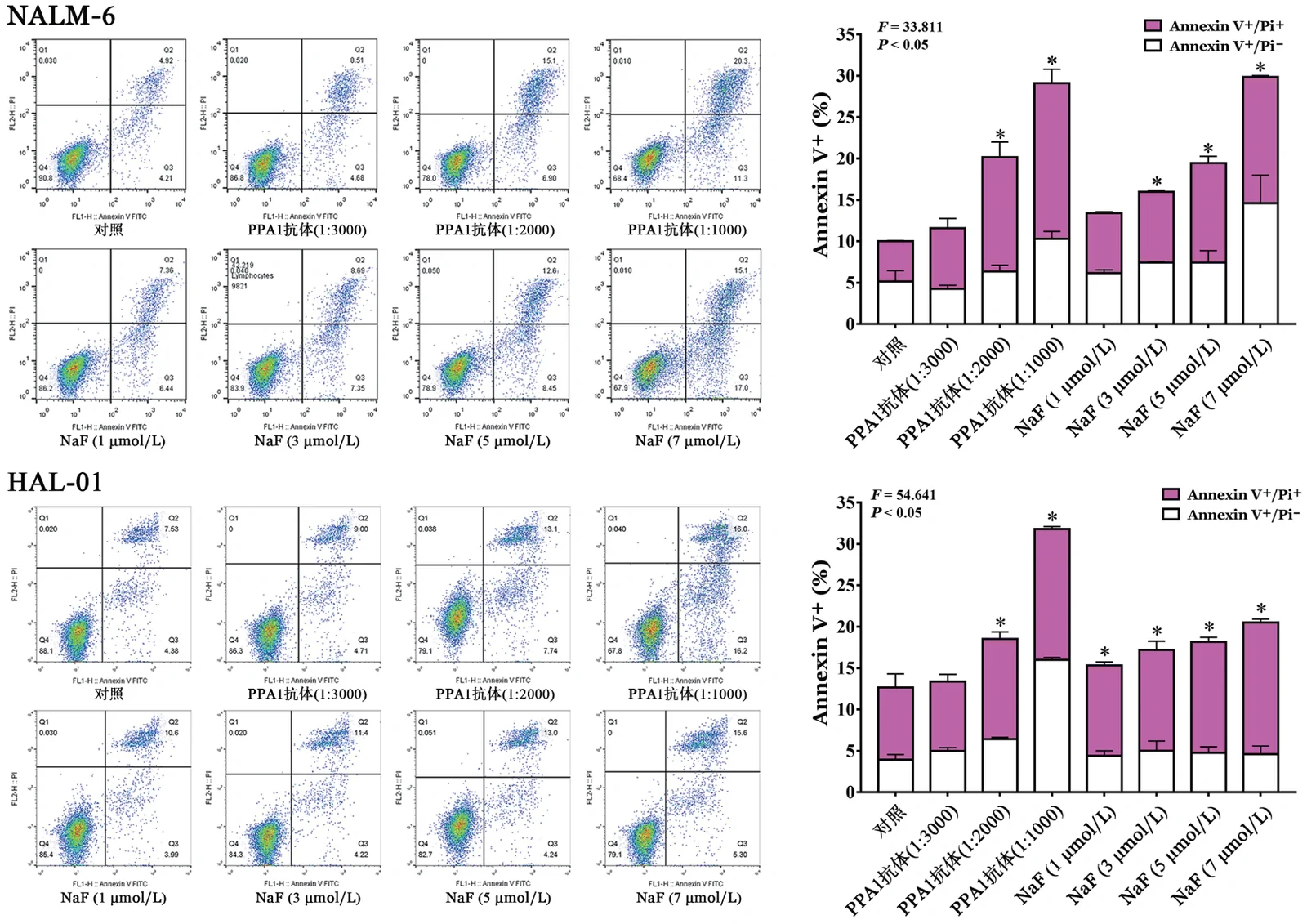
流式细胞术检测PPA1单抗、NaF对NALM-6、HAL-01细胞凋亡的促进作用（实验重复3次，与对照组比较，*P<0.05）

7. PPA1单抗对NALM-6与HAL-01细胞线粒体膜电位及细胞周期的影响：流式细胞术检测显示，PPA1单抗处理可显著升高NALM-6和HAL-01细胞中JC-1单体比例。与对照组相比，1∶2 000和1∶1 000稀释的PPA1单抗处理后，NALM-6细胞JC-1单体比例分别增至对照组的4.08倍和6.49倍；同样条件下，HAL-01细胞JC-1单体比例分别增至对照组的3.99倍和6.42倍，差异均具有统计学意义（*F*_NALM-6_＝357.50，*F*_HAL-01_＝614.71，*P*<0.05），提示PPA1单抗可降低NALM-6和HAL-01细胞线粒体膜电位（图6A）。细胞周期检测结果显示，PPA1单抗处理可使NALM-6和HAL-01细胞周期阻滞于G_0_/G_1_期。与对照组相比，经1∶2 000和1∶1 000稀释的PPA1单抗处理后，NALM-6细胞G_0_/G_1_期比例由76.05％分别升高至83.40％和91.22％；HAL-01细胞G_0_/G_1_期比例由72.24％分别升高至81.70％和89.15％（*P*<0.05），S期和G_2_期细胞比例相应降低（图6B）。

**图6:**
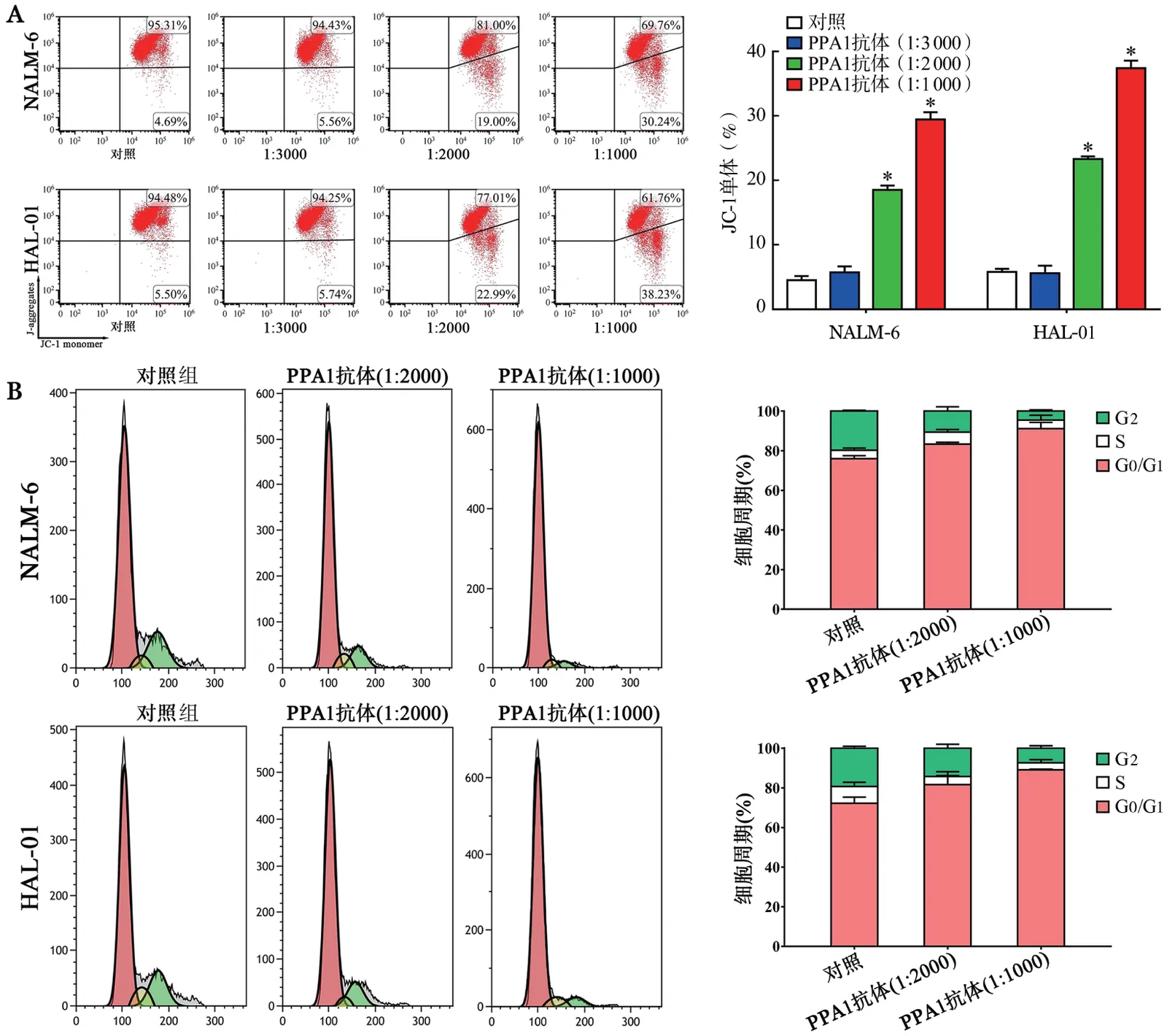
PPA1单抗对NALM-6与HAL-01细胞线粒体膜电位及细胞周期的影响（实验重复3次，与对照组比较，*P<0.05） **A** 线粒体膜电位检测；**B** 细胞周期检测

8. PPA1过表达对细胞增殖的促进作用：qPCR检测结果显示，与空白对照及GV492对照组比较，GV492-PPA1细胞组中PPA1 mRNA水平显著升高，分别是对照组的2.32和1.84倍（图7A），差异具有统计学意义（*F*_NALM-6_＝26.79，*F*_HAL-01_＝31.76，*P*<0.05）。Western blot检测各组细胞PPA1蛋白表达水平，Image J灰度分析结果显示GV492-PPA1组NALM-6和HAL-01细胞PPA1蛋白表达水平增高，是未处理组的2.03和1.76倍（图7B、C）（*F*_NALM-6_＝68.97，*F*_HAL-01_＝57.51，*P*<0.05）。CCK-8结果显示，培养48和72 h后，GV492-PPA1组NALM-6细胞增殖能力分别上调25.05％和26.14％，HAL-01细胞增殖率分别上调26.14％和29.25％（图7D、E）（*P*<0.05），表明PPA1过表达可促进肿瘤细胞增殖。

**图7:**
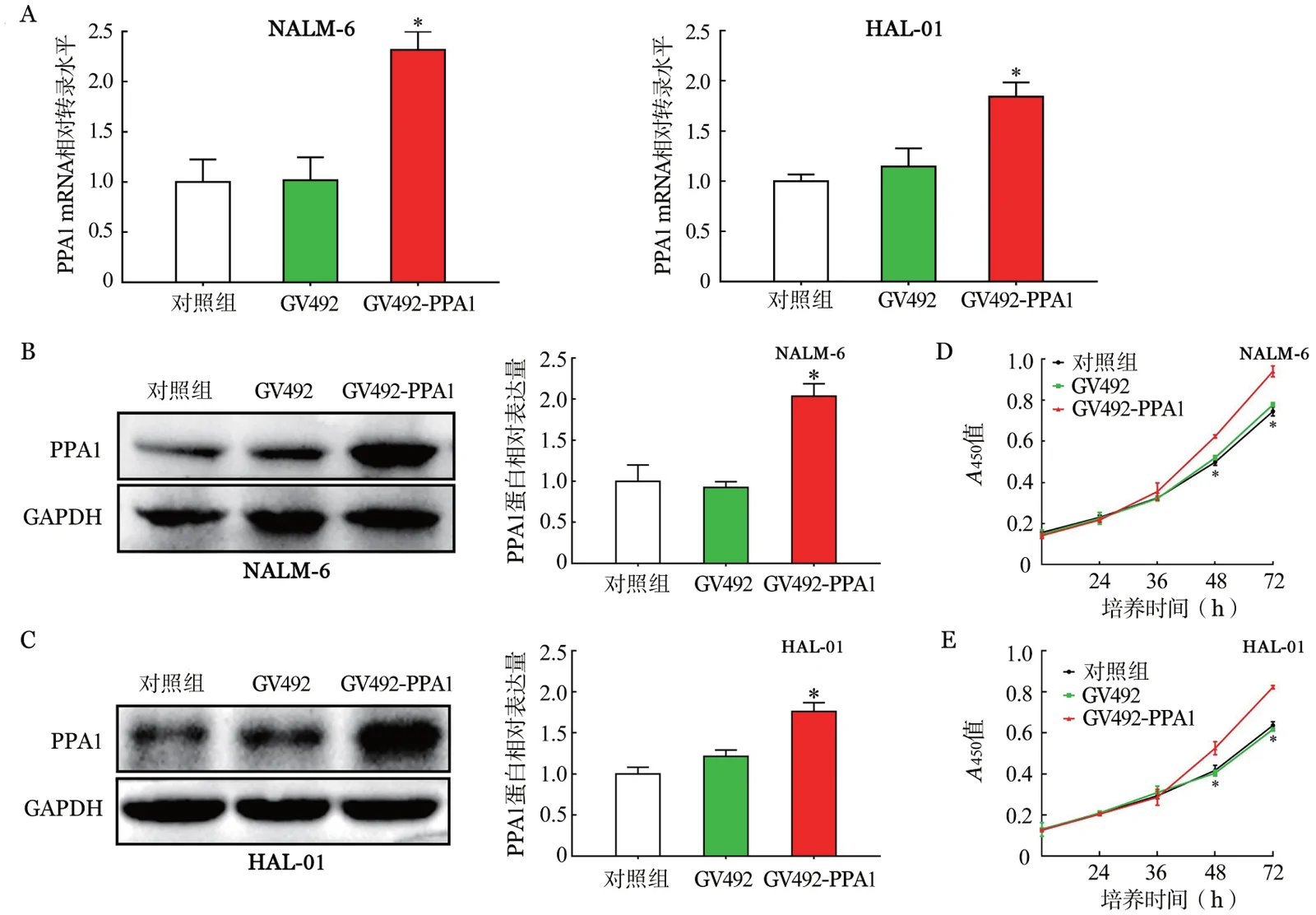
qPCR和Western blot检测PPA1过表达及其对细胞增殖的影响（实验重复3次，与对照组比较，*P<0.05） **A** qPCR检测PPA1 mRNA转录水平；**B、C** Western blot检测PPA1蛋白表达水平；**D、E** PPA1过表达对NALM-6和HAL-01细胞增殖的影响

9. 抑制PPA1表达对细胞增殖的抑制作用：慢病毒感染后，qPCR和Western blot检测结果显示，GV644-shPPA1组NALM-6和HAL-01细胞中PPA1的mRNA转录水平（抑制率分别为58.97％和45.99％）及蛋白表达水平（抑制率分别为36.85％和27.02％）均显著低于未处理组和GV644对照组（*P*<0.05）（图8A～C）。CCK-8结果显示，与对照组相比，GV644-shPPA1组NALM-6细胞在48 h和72 h的增殖抑制率分别为19.77％和28.99％（*P*<0.05），HAL-01细胞在72 h的增殖抑制率为20.90％（*P*<0.05）（图8D、E）。

**图8:**
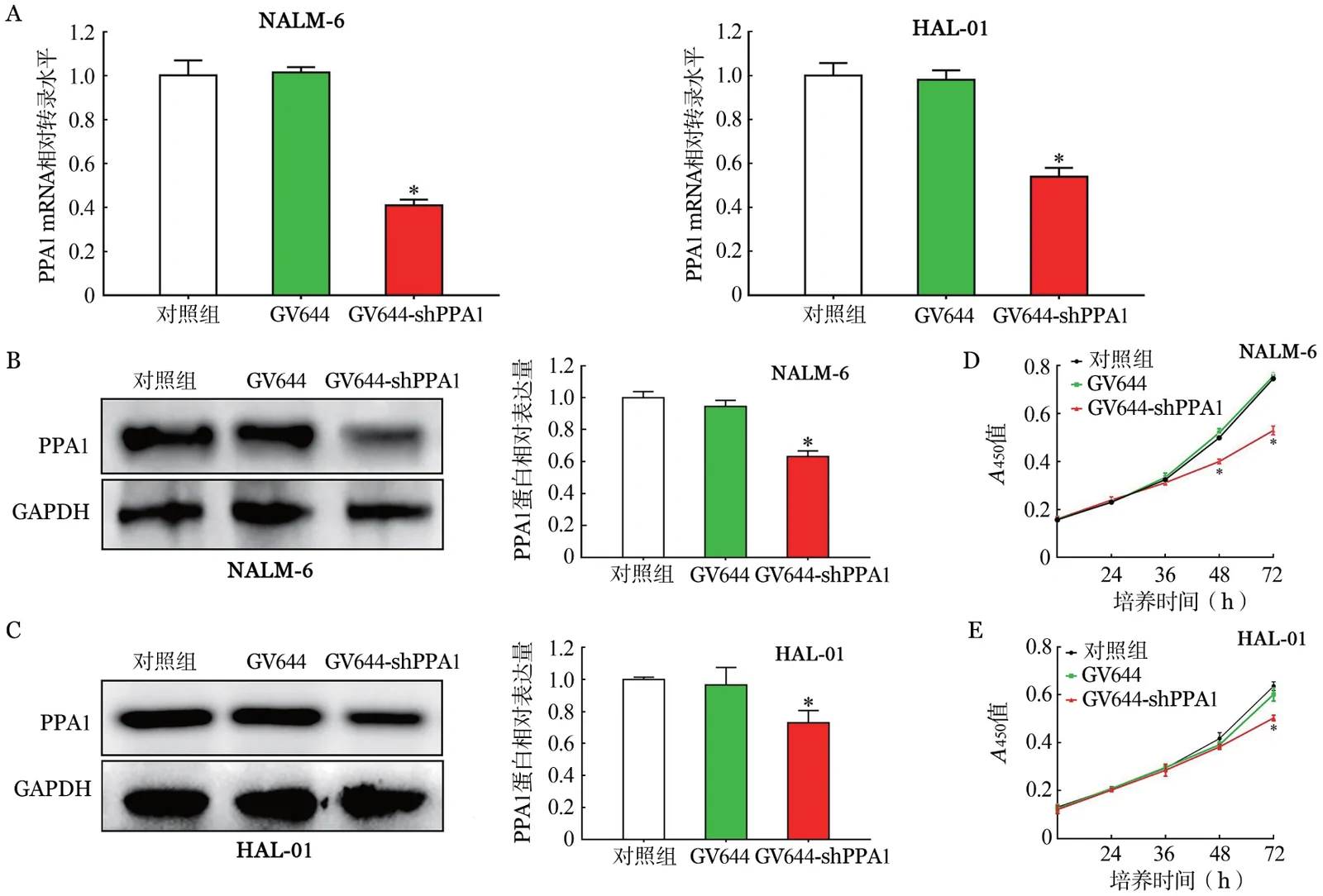
shRNA抑制PPA1表达及其对NALM-6和HAL-01细胞增殖的影响（实验重复3次，与对照组比较，*P<0.05） **A** qPCR检测PPA1 mRNA转录水平；**B、C** Western blot检测PPA1蛋白表达水平；**D、E** 抑制PPA1表达对NALM-6和HAL-01细胞增殖的影响

10. 抑制PPA1表达对PI3K/AKT通路磷酸化的抑制作用：Western blot结果显示，沉默PPA1可显著抑制PI3K/AKT信号通路的激活。如图9所示，与对照组相比，敲低PPA1后，NALM-6细胞中p-PI3K/PI3K及p-AKT/AKT比值分别下降29.73％和68.58％，HAL-01细胞中分别下降38.34％和25.84％（*P*<0.05），提示PI3K/AKT通路磷酸化受抑制。同时，MDM2表达下调（NALM-6和HAL-01抑制率分别为67.78％和55.56％），P53表达上调（分别为对照组的1.73倍和2.46倍）（*P*<0.05）。凋亡相关因子检测（图10）显示，NALM-6与HAL-01细胞Bcl-2表达下调（抑制率分别为50.85％和55.82％），Bax（分别为对照组的1.49倍和1.56倍）及Cleaved Caspase-3（分别为1.34倍和1.68倍）表达上调（*P*<0.05）。

**图9:**
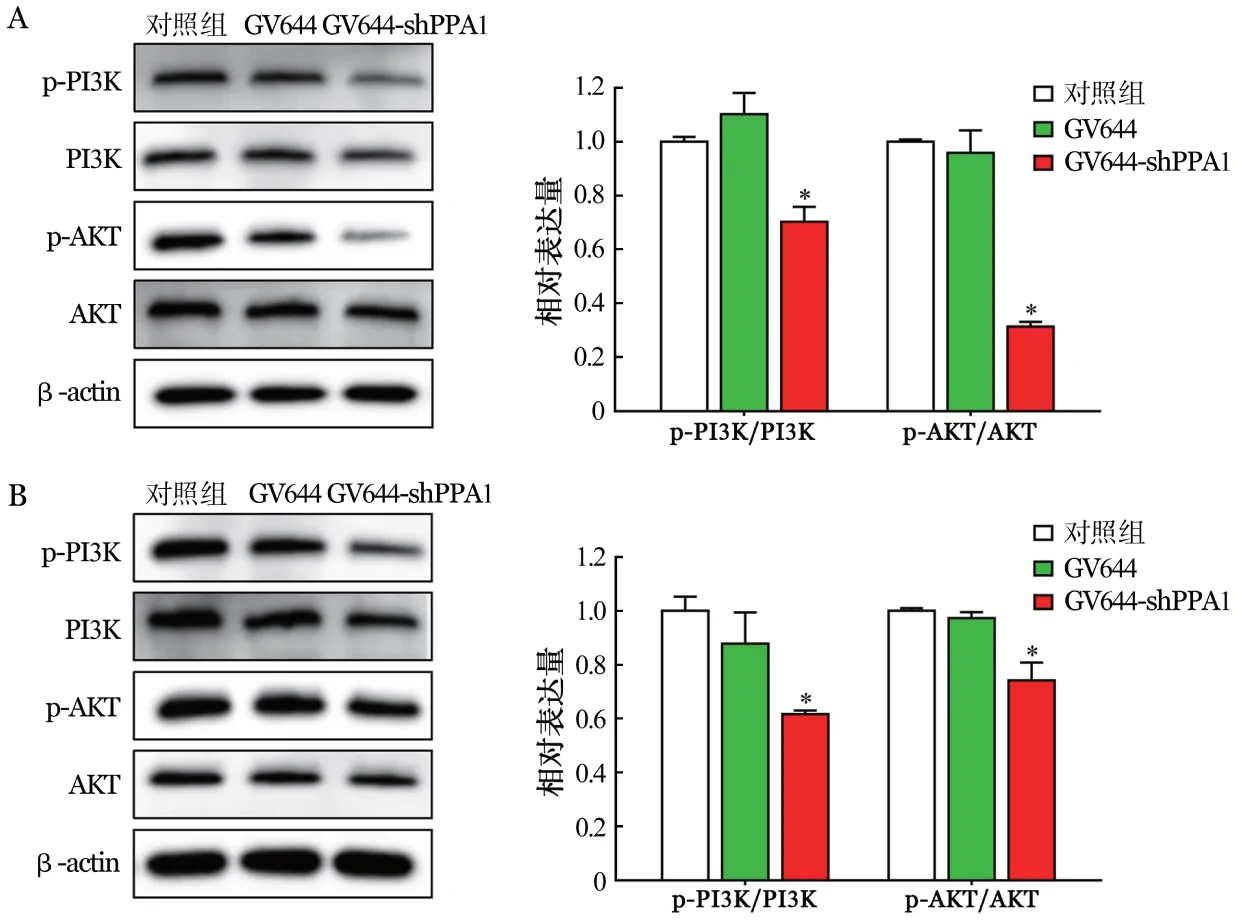
shRNA抑制PPA1表达对PI3K/AKT磷酸化的抑制作用（实验重复3次，与对照组比较，*P<0.05） **A** Western blot检测NALM-6细胞PI3K/AKT通路相关蛋白；**B** Western blot检测HAL-01细胞PI3K/AKT通路相关蛋白

**图10:**
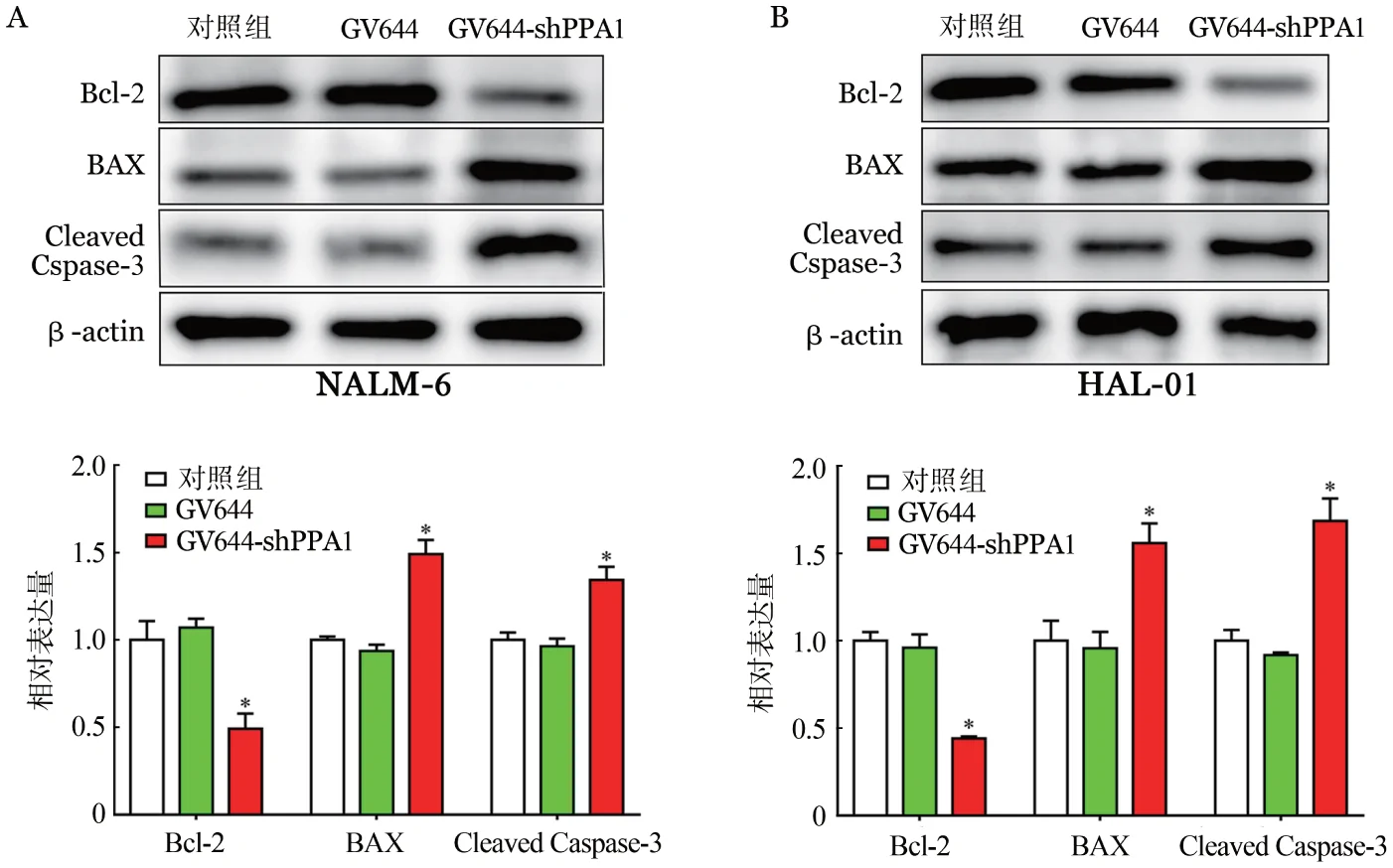
shRNA抑制PPA1表达后凋亡相关因子的检测（实验重复3次，与对照组比较，*P<0.05） **A** Western blot检测NALM-6细胞凋亡相关因子；**B** Western blot检测HAL-01细胞凋亡相关因子

## 讨论

在遗传水平上，B-ALL是一种异质性疾病，其发生发展通常伴随着染色体异常、基因突变和DNA拷贝数量的改变[17]。随着测序技术的进步，越来越多肿瘤相关基因靶点被发现，且这些基因异常已用作诊断、预后和预测性生物标志物，在疾病早期筛查、风险分层和治疗中发挥重要作用[18]。探索新的B-ALL生物标志物并阐明其生物学特性，有助于更精准地监测疾病进展并优化临床诊治策略。

肿瘤细胞快速增殖对能量的高需求及生长调控异常，可能导致PPA1在肿瘤细胞中呈高表达状态[19]。实体瘤相关研究表明，PPA1在胃肠道癌、肺癌和卵巢癌肿瘤中均存在高表达，提示其在肿瘤发生发展过程中起着关键调控作用[20]–[22]。另有研究通过对心肌病患者进行转录组测序分析，证实PPA1突变具有强致病性，并表示PPA1可能是一种心肌病相关蛋白[23]。上述研究均表明PPA1具有作为疾病相关标志物的潜力，但目前PPA1在血液系统相关疾病中大的作用及机制尚不清晰，其对B-ALL异常幼稚淋巴细胞增殖和凋亡的影响尚未见报道。

焦磷酸酶主要分为两大类，为可溶性和膜整合性焦磷酸酶[24]。PPA1属于典型的可溶性焦磷酸酶，本研究选取PPA1为研究对象，发现PPA1在B-ALL患者异常幼稚淋巴细胞中转录及表达水平显著升高。体外实验显示，rPPA1蛋白可促进NALM-6和HAL-01细胞增殖，而PPA1单抗及慢病毒介导的基因敲低则抑制增殖、阻滞细胞周期于G_0_/G_1_期、并诱导细胞凋亡。机制上，抑制PPA1可下调PI3K/AKT通路磷酸化，进而阻碍MDM2入核，减少P53泛素化降解，同时上调Bax、下调Bcl-2并激活Caspase-3，最终协同抑制增殖、促进凋亡。

值得注意的是，除上述信号通路调控外，PPA1在B-ALL细胞代谢重编程中亦可能扮演关键角色。PPA1的核心生化功能是催化焦磷酸盐（PPi）水解为正磷酸盐（Pi），而Pi是合成ATP、核酸及磷脂的必需原料，直接参与细胞能量代谢与生物合成[25]。白血病细胞为满足快速增殖需求，常发生代谢重编程，如增强糖酵解与核苷酸合成。本研究中PPA1的高表达，可能正是为了维持细胞内高水平的Pi库，从而支持ATP的持续生成、核酸的快速合成以及膜磷脂的组装，为细胞增殖提供代谢基础。抑制PPA1后，细胞内Pi/PPi平衡被打破，可能导致ATP与生物合成前体供应相对不足，进而通过能量感应通路（如AMPK）或底物限制，间接影响PI3K/AKT等生长信号通路的活性，最终抑制增殖并诱导凋亡[26]。因此，PPA1可能作为连接细胞代谢状态与增殖/凋亡信号调控的关键分子，其高表达或可视为B-ALL代谢重编程的重要特征之一。未来可通过代谢组学等技术，进一步解析PPA1如何精准调控白血病细胞能量与合成代谢，更全面阐明其在B-ALL发生发展中的功能。

需要指出的是，本研究存在一定局限性。由于仅纳入初治B-ALL患者新鲜骨髓标本，并经磁珠分选获取高纯度幼稚B淋巴细胞，严格的入组与排除标准导致病例数有限，样本量相对较小。尽管本研究中PPA1在患者与正常对照间表达差异的效应量（Cohen's d＝0.749）属于中等偏高水平，提示存在较强的生物学信号，且在高度同质的队列中提升了结果的内部可靠性。然而，较小的样本量仍可能降低统计功效，影响对微弱效应值的检出。因此，本研究后续将通过多中心、大样本的独立队列进一步验证PPA1在B-ALL中的作用及临床意义，并在不同疾病亚型中进行深入探索。

## Supplementary Material


